# Analysis of gene expression dynamics revealed delayed and abnormal epidermal repair process in aged compared to young skin

**DOI:** 10.1007/s00403-015-1551-5

**Published:** 2015-03-06

**Authors:** Peggy Sextius, Claire Marionnet, Charlotte Tacheau, François-Xavier Bon, Philippe Bastien, Alain Mauviel, Bruno A. Bernard, Françoise Bernerd, Louis Dubertret

**Affiliations:** 1 L’Oréal Research and Innovation, 1, avenue Eugène Schueller, 93600 Aulnay Sous Bois, France; 2 ARIC, Hôpital Saint Louis, Paris, France; 3 Polyclinique de Dermatologie, Hôpital Saint Louis, Paris, France; 4 Institut Curie, Orsay, France

**Keywords:** Epidermis, Repair, Gene expression, Aging, Microarray

## Abstract

With aging, epidermal homeostasis and barrier function are disrupted. In a previous study, we analyzed the transcriptomic response of young skin epidermis after stratum corneum removal, and obtained a global kinetic view of the molecular processes involved in barrier function recovery. In the present study, the same analysis was performed in aged skin in order to better understand the defects which occur with aging. Thirty healthy male volunteers (67 ± 4 years old) were involved. Tape-strippings were carried out on the inner face of one forearm, the other unstripped forearm serving as control. At 2, 6, 18, 30 and 72 h after stripping, TEWL measurements were taken, and epidermis samples were collected. Total RNA was extracted and analyzed using DermArray^®^ cDNA microarrays. The results highlighted that barrier function recovery and overall kinetics of gene expression were delayed following stripping in aged skin. Indeed, the TEWL measurements showed that barrier recovery in the young group appeared to be dramatically significant during the overall kinetics, while there were no significant evolution in the aged group until 30 h. Moreover, gene expression analysis revealed that the number of modulated genes following tape stripping increased as a function of time and reached a peak at 6 h after tape stripping in young skin, while it was at 30 h in aged skin, showing that cellular activity linked to the repair process may be engaged earlier in young epidermis than in aged epidermis. A total of 370 genes were modulated in the young group. In the aged group, 382 genes were modulated, whose 184 were also modulated in the young group. Only eight genes that were modulated in both groups were significantly differently modulated. The characterization of these genes into 15 functional families helped to draw a scenario for the aging process affecting epidermal repair capacity.

## Introduction

Besides the obvious consequences of aging on skin appearance (wrinkles, sagging, loss of elasticity, dyschromia), some discomforts have been reported, including increased susceptibility to irritants, contact dermatitis and severe xerosis, which are likely linked to altered epidermal barrier permeability and epidermal homeostasis. Indeed, despite normal thickness of the stratum corneum (SC) [12] and minor differences in barrier function [10], a higher prevalence of chronic xerosis is frequently observed in aged subjects, with increased trans-epidermal water loss (TEWL). In fact, intercellular lipid composition in aging SC is decreased or altered, especially during winter time [20, 22]. These changes in SC composition alter its physical–chemical properties with respect to barrier function. SC becomes notably more sensitive to physical or chemical aggressions like tape-stripping (TS) or acetone, while the permeation of hydrophilic drugs is decreased [21]. Furthermore, although an age-dependent decline of overall positive patch tests is observed, the seriousness of contact allergies is increased, which may be due to a slower epidermal turn-over [17]. Lastly, barrier function recovery after SC removal slows down with age, which is most likely to be attributable to epidermal functional abnormalities [5, 10, 11].

Until now, most of the reported clinical studies used TEWL measurements to ascertain the effect of drug treatments on skin barrier function or epidermal homeostasis recovery after aggression. In previous studies, we highlighted the interest of performing transcriptional analysis to further characterize the effects of different physiological aggressions or cosmetic treatments on human epidermal barrier function [13], and we investigated the dynamics of epidermal repair after SC removal in young subjects using TEWL measurements and cDNA microarray analysis at five time points [23]. TEWL measurements gave a macroscopic view of the kinetics of barrier recovery whilst microarray analysis chronologically identified the main molecular processes that take place during epidermal recovery.

In the present study, we applied this approach to characterize barrier function recovery in aged subjects, with the aim of identifying pertinent, reproducible and significant markers, which would reflect an age-related impairment of barrier function.

## Materials and methods

### SC removal

30 healthy male caucasian volunteers aged 67 ± 4 years with phototype II or III were included in the study. They had no history of dermatological disorders, skin allergies, nor hormonal or vitamin treatments. The study was conducted according to the Helsinki declaration. All volunteers gave informed consent. The study was approved by the bio-ethics committees of Saint-Louis and Boucicaut Hospitals, Paris, France. The present study follows a previous similar study in which a set of 30 young volunteers whose mean age was 27 ± 4 years had been included [21].

In both populations, SC removal was performed using sequential adhesive tape strips of the inner forearm skin on the test area until skin glistened [18, 23]. On average, 48 ± 7 strips were performed in the aged group and 45 ± 8 strips in the young group to obtain the expected total stratum corneum removal [18, 23]. The skin of the other inner forearm was used as a control.

### Tissue collection

Epidermis samples (1.5 × 1.5 cm) of stripped and control skin were removed under local anesthesia, using a dermatome GA630 (AESCULAP, Melsungen, Germany). Five sub-groups of six volunteers were randomly set in both populations. Tissue samples were collected from both forearms at same time points following stripping per subgroup (2, 6, 18, 30 or 72 h, respectively). RNA were extracted and stored as described by Sextius et al. [23].

### Kinetics of barrier recovery

TEWL was measured using an EP1 evaporimeter (Servomed, Kinna, Sweden) before, immediately after stripping, and before tissue collection on both stripped and control forearms. The measurements were done in a dedicated room with controlled temperature (21 ± 2 °C) and hygrometry (45 ± 5 %). The kinetics of barrier function recovery was assessed by calculating the rate of barrier recovery (%BR) at each time point. %BR = 100 × [1 − (*c* − *a*)/(*b* − *a*)] where* a*,* b*,* c* denote TEWL before stripping, TEWL immediately after stripping, and TEWL at each time point after stripping, respectively. The evolution of TEWL recovery over time has been tested in each age group using the non-parametric Jonckeere–Terpstra trend test. This test allows to test the hypothesis of a monotonic evolution over time. The difference was considered as significant when the *p* values were under 0.05.

### Differential hybridization

Differential hybridization on cDNA microarrays was performed as described by Sextius et al. [23]. Briefly, 2.5 µg of total RNA were used for reverse transcription using ^33^P (Amersham) radiolabed dCTP nucleotides and AMV reverse transcriptase (Invitrogen SARL, Cergy Pontoise, France). DermArray^®^ cDNA microarrays (IntegriDerm, Birmingham, AL, USA) including 4405 unique cDNAs were used for hybridization.

Image quantification and signal correction were done as previously described [23]. A threshold value of signal intensity was determined using an iterative algorithm [2, 7]. The average* A* and the standard deviation SD of background signal was calculated and the threshold value was set at *A* + 3SD. Genes for which both control and stripped signals were lower than the threshold were not taken into account. If one of either signal was lower while the second was higher than the threshold, the threshold value of the weaker signal was used instead.

### Identification of modulated genes

The fold change after stripping was calculated for each gene, by dividing the corrected signals of the stripped samples by those of the control samples. A mean fold change was calculated including the 6 volunteers per time point, at each time point. A gene was considered as modulated at one time point when: (a) the mean ratio at log 2 scale was significantly different from 1 (Student’s *t* test, *p* < 0.05) and (b) within a time point, at least 50 % of the ratios were higher than two while none were under 0.5 for induced genes, or inversely for repressed genes.

### Data analysis

- If a gene was expressed and modulated in both groups, its modulation profile was compared as a function of age by means of a two factors Anova test (*p* < 0.05). The genes that were markedly different according to age or interaction age/time were selected.
- Some specific genes were significantly expressed and modulated in one age group but not in the other one. These genes were not included in the previous comparison. To select the core of them, we selected those whose modulation was the most relevant. For that purpose, we reduced the comparison to 6 and 30 h, because these two time points comprised the highest number of modulated genes in the young and aged group, respectively. A distance was calculated between the two age groups taking into consideration the differences found at 6 and 30 h.

$${\text{Distance young}}/{\text{aged}} = {\text{Abs }}\left[ {{\text{Log2 }}\left( {R^{{{\text{Y}} - 6\;{\text{h}}}} } \right){-}{\text{Log2 }}\left( {R^{{{\text{A}} - 6\;{\text{h}}}} } \right) + {\text{Log2 }}\left( {R^{{{\text{Y}} - 30\;{\text{h}}}} } \right) - {\text{Log2 }}\left( {R^{{{\text{A}} - 30\;{\text{h}}}} } \right)} \right].$$

with *R* ^Y − 6 h^ = mean ratio expression in young people at 6 h, *R* ^A − 6 h^ = mean ratio expression in aged people at 6 h, *R* ^Y − 30 h^ = mean ratio expression in young people at 30 h, *R* ^A − 30 h^ = mean ratio expression in aged people at 30 h

The mean distance (*D*) and standard error of mean (sem^D^) were calculated. The specifically modulated genes in either aged or young group with a distance of at least D + 2sem^D^ were selected as those that showed the greatest difference between the age groups post TS. A Mann–Whitney statistical test was then performed in order to compare the results that were obtained for each selected gene between young and aged group.

### Bibliographic study

A bibliographic study was performed for all of the genes of interest selected using NCI’s Cancer Genome Anatomy Project website (http://cgap.nci.nih.gov). All genes of interest were characterized and dispatched into 15 functional families which we defined.

## Results

### Kinetics of barrier function recovery

**Fig. 1:**
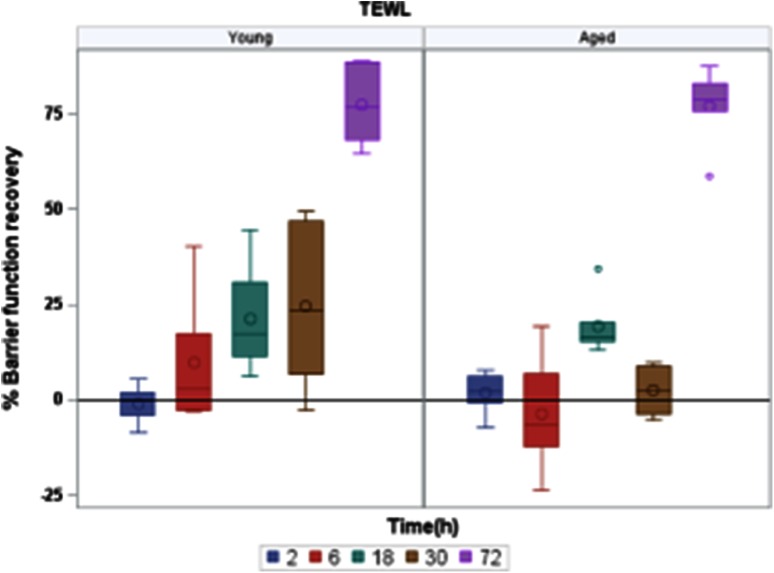
Kinetics of barrier function recovery as assessed by TEWL measurements before, immediately after and 02, 06, 18, 30 and 72 h after TS. A percentage of barrier recovery was calculated at each time after TS. The mean TEWL value that was obtained immediately after SC removal was set to 0 %, and the mean initial TEWL value obtained before aggression was set to 100 %. The evolution of barrier recovery has been displayed using a *boxplot* representation. The *line* and the *circle inside the boxes* represent the median and the mean of barrier function recovery, respectively

SC removal provoked instantly a dramatic increase in TEWL. Then, water loss progressively normalized with time. The percentage of barrier recovery was calculated at each time point following TS, in both age groups, and has been displayed using a boxplot representation (Fig. 1). Its evolution over time has been tested using the non-parametric Jonckeere–Terpstra trend test. Results showed that the recovery in the young group appeared to be dramatically significant (*p* = 0.0011) during the overall kinetics, while there were no significant evolution (*p* = 0.32) in the aged group until 30 h. This result showed that barrier recovery although similar at 72 h, was faster in young than in aged epidermis.

### Identification of modulated genes in young and aged epidermis

382 genes for which expression varied significantly at least once over time following TS were identified in aged epidermis versus 370 in young epidermis [23]. Only 184 genes were modulated in both groups (Table 1). These 184 genes constitute a common transcriptomic signature of the response of epidermis to TS. Besides, 198 genes and 186 genes (Table 1) were only modulated in the aged or in young epidermis, respectively. These two later sets of genes constitute the specific signature of aged and young epidermis in response to TS.

**Table 1:** Functional characterization of genes whose expression was modulated following tape stripping

Main functional groups	Description	*n* (Y)	*n* (A)	Modulated genes (gene symbols)		
				186 specific for young group	184 common to young and aged group	198 specific for aged group
1. Transport	Movement and regulation of proteins into, out of, within, between cells	15	11	SLC16A1; SNX2; SUOX; SYT1; ARF1; RAB5A; ARL4D; SLC6A1; KLHL32	KPNB1; SULT1C2; RAB7A; ARF1; RAB1A; ARF4	AP2S1; SEC61B; NGB; ABCB8; SERPINA3
2. Adhesion	Attachment/regulation of a cell to another cell or to extracellular matrix	17	16	DSC2; HAPLN1; EMB; CD44; ADAM15; CDH3; PXN; ITGB5; PTPRF; CDH1; CTNNA1	SDC4; PTK7; ARHGDIA; GJB2; POSTN; ITGA6	CNTNAP1; IL8; ITGA3; ITGAE; ITGB1BP1; ICAM3; LGALS2; CDH8; ADRM1
3. Detoxification–oxidative stress	Processes that reduce or remove toxicity, response to oxidative stress	14	20	GLRX; GSTM5	SLC25A5; ATP5I; PRNP; HSP90B1; RPS6; MT1E; ATP5O; NDUFV2; GSTP1; HSP90AB1; SDHA	UQCRQ; GSTA4; ATP5F1; ATP5C1; GPX3; ATP5D; NDUFB9; ETFA
4. Cellular matrix	Cellular matrix constituent	19	15	SPTA1; KIF3C; PLEC1; LMNA; ARPC3; TUBB3; GSN; ARPC3; TUBB4	CBX3; TMSB4X; TUBA3C; LOC643224; TUBA1B; TUBB2A; ACTR3; ACTB; LAD1	TUBA3C; ARPC1A; PFN1; ARPC4; ARPC2
5. Immune response	Processes related to immune response, to immune system and regulation	20	19	PTGES; MLF2; ABCF1; LILRB2; THBD; CTSE; LCP2; NCDN; CD55; ICAM1	S100A8; YBX1; TMSB4X; PPIC; AZGP1P1; IFITM2; AZGP1; S100A9; CD59; ANXA1	BCRP1(BANF1, ABCG2); IFI17; IFI27; CLEC2B; IFNGR2; ANXA5; D4S234E; CD36; HAX1
6. Communication	Processes that mediate interactions between a cell and its surroundings, cell signaling	33	25	RGS4; KDR; PRKCH; TBL3; ZFP36L1(BRF1); EFNB1; DLGAP4; PRKCB1; IFNAR2; VRK3; PTK2; GLG1; YWHAE; DIO3; NR4A1; MAPK10; GNAO1; GNAZ; EPHB2	CRHBP; S100A10; EPHB3; S100A2; GJA1; CSNK1A1; PTGES3; FOSL1; DUSP7; CALM2; PTP4A2; JUNB; SIGMAR1	GNAI2; PIK3CD; TRAP1; PTP4A2; RUNDC3A; IRAK1; TLE4; MAP2K3; EFNA1; PRKCG; HINT1; MAPKAPK3
7. Cell cycle growth–proliferation	Cell replication, increase in size, expansion of the cell population, cell division	37	32	FLT1; HBEGF; PLAU; BST2; EGR1; CCNG2; IFI16; IGFBP3; SLC3A2; VAT1; KHDRBS1; MAPK6; CDK6; NDUFA5; PMP22; CAP1; TSPAN31; SYK; HBEGF; CXCL1	BZW1; HDGF; KLK10; MYC; PPM1G; PA2G4; YWHAQ; NME1; NPM1; CAPNS1; PRDX1; AREG; FGFBP1; SEPT2; KLF4; FOSB	TCF3; TSPAN-3; UBE2C; PCNA; CDC37; DDX5; EWSR1; LMO2; IGFBP6; ISG20; ARPC2; SOCS1; GSPT1; MYL4; PHB; CDK2AP1
8. DNA–RNA–transcription–translation	DNA synthesis, repair, amplification, RNA processing, splicing-transcription and translation processing and regulation	72	70	RBM3; ST18; SAP18; EIF4E; MEF2A; IARS; PLAG1; XRCC1; METAP2; NRAS; PCBP1; RNPS1; CRYAB; DDB1; SSX3; RPL24; FBL; HMGB1; PAR5; SF3B4; H2AFB2; MEF2D; LOC646171; PURA; ZFP36; EGR3; CHAF1B; HARS; CDC27	EIF2S2; KLF6; SUB1; HNRPA1; SNRPG; SFRS2; SF1; RPP30; EIF1; NCL; SRPR; SNRPB; CACNA1I; H3F3B; SET; LDHB; GARS; TRIM28; SNRPF; XRCC5; CCT6A; POLR2L; JUNB; JUNB; HNRNPA1; EBNA1BP2; EIF2S2; CCT3; SFRS1; HNRPA2B1; HNRNPABHNRNPA1; EBNA1BP2; EIF2S2; CCT3; SFRS1; HNRPA2B1; HNRNPABNOP56; HNRNPC; CCT6A; EIF4A1; SNRPD1; BGN; EIF4A3; NCL; JUNB; HNRNPA1; EBNA1BP2; EIF2S2; CCT3; SFRS1; HNRPA2B1; HNRNPAB	SNRPA1; SFRS6; SYNCRIP; PPIF; BUD31; POLR2F; SRP72; POLR2G; ZNF212; KDELR3; KARS; HNRPR; SFRS4; LRRFIP1(EPRS); RPA1; TCEB2; SF3B2; NASP; EIF3B; PTBP1; HNF1B; EIF4EBP2 (SSBP3); HIST1H4C; TRAB2; POLR2D; SSBP1; ARD1A; BTF3
9. Proteolysis	Hydrolysis of a peptide bond or bonds within a protein	20	25	UBE2D2; PSMD8; UBA1; METAP2; UBA1; SERPINB8	PSMC1; SERPINB3; PSMD2; PSMA3; PSME3; PSMB7; RPL32; PSMB5; P11; PSMD2; CSTB; PSMB1; MMP3; CSTA	ECE1; PSMC3; TRB@; PSMD6; USP10; COPS3; PSMA5; PSMB3; PSMA7; PSME2; PGA3
10. Energy	Processes involved in the liberation of energy	23	26	PCK1; SMPD1; COX8A; CYP2S1; ATP2A2; DLD; ABCE1	COX6A1; PGK1; PGAM1; TPI1; CYC1(CYCS); ALDOA; NDUFA11; LDHA; GAPDH; COX6B1; DPYS; CHST11; COMT; CYC1(CYCS); SULT2B1	NDUFB4; NDUFS1; CYP3A4; CYC1; LDHC; COX7C; COX7B; BPGM; CA2; QDPR; COX6C
11. Cell differentiation	Acquisition of specialized structural and/or the functional features that characterize the mature cells	20	22	EVPL; KRT20; NDRG1; ECM1	KRT6B; KRT5; SPRR2C; SPRR1B; 0SPINT1; KRT10; S100A7; KRT16; KRT8; KRT4; KRT18; FLG; IVL; KRT14; KRT13	INHBB; KRT7; KRT1; TGM1; KRT13
12. Lipids (barrier function)	Synthesis, metabolism and processing of the lipids of lamellar bodies or involved in barrier function	5	4	PLCL1; ADFP; CTSA; FDPS	ACAT2	GM2A; HSD17B10; NSUN4
13. Apoptosis	Genes related to apoptosis and regulation of apoptosis	6	11	API5L1; DAD1; TMBIM6; SST; NFKBIA	SERPINB2	TIMP3; MAP3K5; PDCD5; APP; EI24; PDCD6; IL2RA; DAPK3; BAK1; DNASE1L3
14. Diverse (weakly represented functional groups : contain <5 genes)	Apoptosis, amino acid metabolism, melanin synthesis, Golgi apparatus, development, lipid metabolism, extracellular matrix, ion transport, nucleotide synthesis	26	36	HPD; MMP7; SLC31A1; GOLGA4; BMP4; DRG1; VSNL1; DCT; PAICS; CACNA2D2; PMEL; SST; BGN	MMP1; TYRP1; P4HB; ARG1; ODC1; CLIC1; MYL12A; OAZ1; NME2; SLC4A3; SRM; MT1L; P4HB; CALU; SERPINB2	FXYD3; CANX; TSTA3; ROR1; PRRG2; KTN1; CRIP2; GOT2; COL1A2; APRT; APP; COL6A2; ANXA6; ATP1A1; PTS; MT1G; MT1B; MT1H; MT1F; IMPDH1; CTBP1; HAL
15. Unknown	Expressed genes with undefined ontology	43	50	FAM83A; ANXA8L1; HNRNPL; JMJD4; ITGAM; FAM82B; IFT140; FAM160A2; ARL4D; IGHM; NAPEPLD; NCDN; TIMP1; EIF1; SAT1; TOP1; CYC1; IL3RA; ZNF740	TREML2; RCVRN; NAMPT; GNL2; HNRNPA3; FAM129B; PTMA	ATXN2L; SART1; ESRRA; ALDH18A1; AURKAIP1; PTCH1; CCDC40; PSMC6; SSSCA1; HN1; DDR1; MCRS1; CD99L2; NQO2; FCN1; IGHV5-78; NID1; SUMO2; COX16; FGA; TUBG1; CD52(TMEM126A), TMEM50A; TAGLN2; SYNPO; PPY2; ITPKC; RPS29; EWSR1; FGF2; PLAC2; MT1F; TUBG1; CD52(TMEM126A)
	Total	370	382			

*N* number of modulated genes, *Y* young group, *A* aged group

### Kinetics of gene expression during epidermal repair of young and aged epidermis

**Fig. 2:**
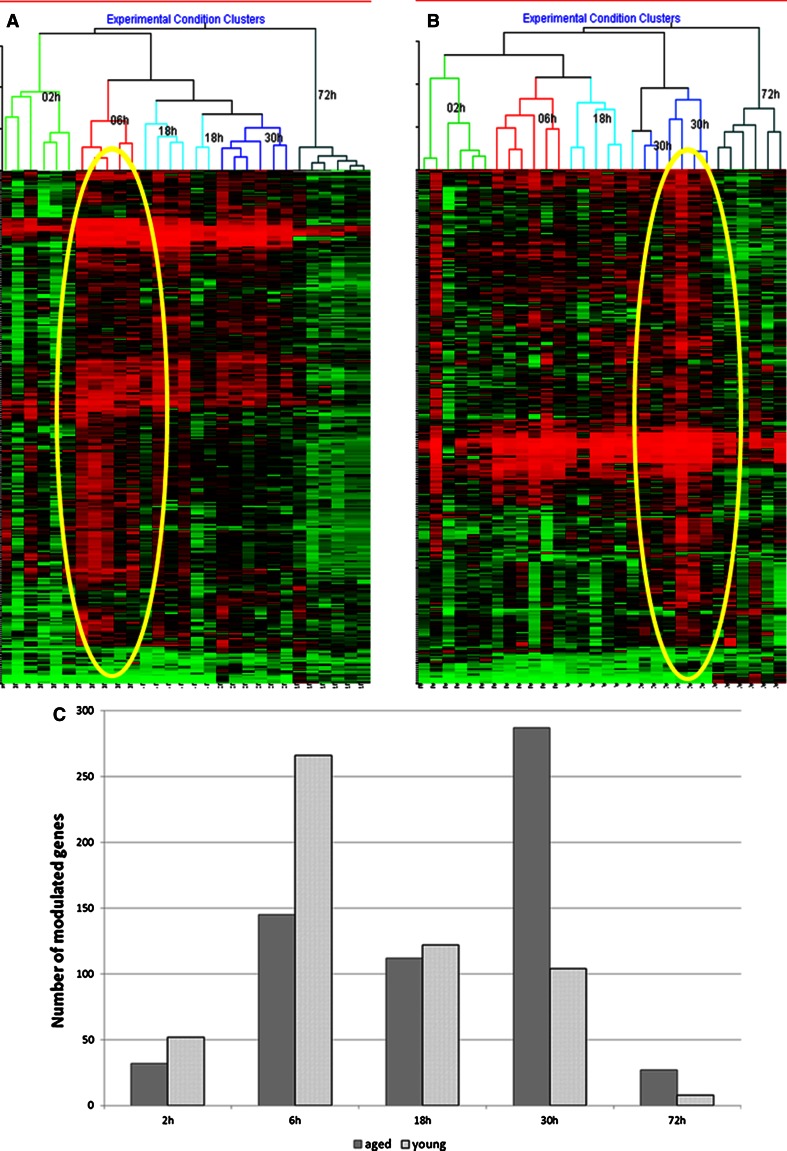
Characterization of gene expression during epidermal repair in young and aged epidermis. The 370 modulated genes in young epidermis and 382 modulated genes in aged epidermis were classified by using (1) the hierarchical clustering method (**a** young epidermis, **b** aged epidermis); (2) a kinetic distribution graphic (**c**)

Six volunteers were included per time point and 5 time points were studied. This gave 30 subsets of 370 and 382 modulated genes in the young and aged group, respectively. The whole fold change values corresponding to these modulated genes were gathered and classified using a non-supervised hierarchical clustering aiming to analyze the data without a priori. This classification method gathered the data as a function of the time group the volunteers belonged to (Fig. 2a, b). One objective of the study was to follow gene expression as a function of time in a young and an aged group of volunteers. If several factors could influence the data (interindividual variability, technical variability, etc.), this clustering showed that time is the major contributing factor that influenced the gene expression data in this study, despite the limited number of volunteers per time points. Individual variability in such dynamics response seemed to be very low in comparison with time influence on gene expression following TS.

Figure 2 also shows that the kinetics of gene expression is different between aged and young groups. The more intense variations in gene expression were clearly found at 6 h in the young group and at 30 h in the aged group. Indeed, in young epidermis the number of modulated genes increased dramatically from 52 at 2 h to 266 at 6 h and decreased progressively to 122 at 18 h, 104 at 30 h and 8 at 72 h (Fig. 2c). In aged epidermis, 32 genes were modulated at 2 h and 145 and 112 at 6 and 18 h, respectively. The number of modulated genes in aged epidermis dramatically increased to 286 at 30 h and 27 genes were still modulated at 72 h.

### Functional analysis of modulated genes

Modulated genes were gathered together into 15 functional families [23], and classified as a function of the time when they were modulated (Table 2).

**Table 2:**
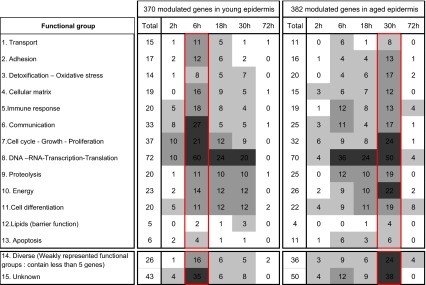
Time distribution of modulated genes in young and aged skin as a function of the functional group they belong to

The shading darkens as the number of modulated genes increases

The functional analysis showed no obvious differences in the molecular functions of the modulated genes between aged or young groups. The number of modulated genes in each functional family was also similar. The main difference was related to the time when each biological function was involved during barrier recovery that appeared to be overall delayed in aged group. Genes involved in cell cycle, cell growth and proliferation, or in DNA, RNA and protein processing family were two representative examples (Table 2). The distribution of these genes as a function of time followed a bell-shaped curve which peaked at 6 h in young epidermis and 30 h in aged epidermis.

### Overall comparison of modulated genes between young and aged epidermis

184 genes whose expression varied at least once over time in both age groups were selected. Their profiles were compared by performing a two way Anova test. This allowed determining the genes whose expression varied significantly as a function of time, as a function of the age of the volunteer and also those for whom the age of the donor influenced the time when the modulation occurred. The two last subgroups (age and interaction age/time) were particularly interesting since they tell about the differences in gene modulation as a function of age.

**Fig. 3:**
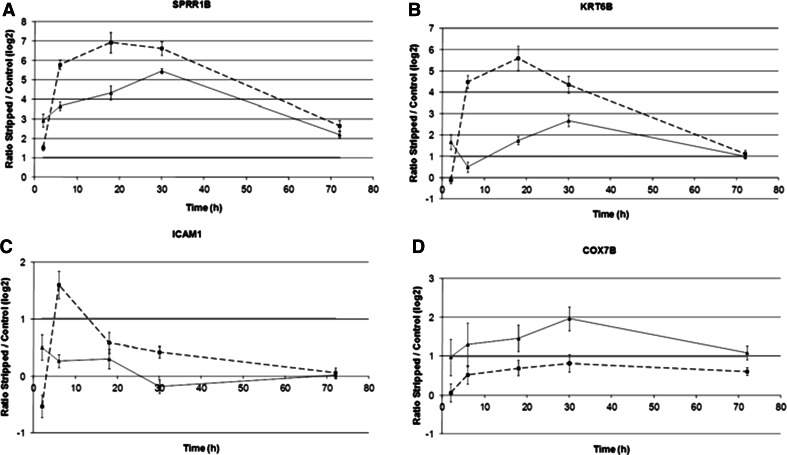
Example of four genes whose expression profiles after TS were significantly different between young and aged epidermis. The *curves* represent modulations of gene expression at log2 scale as a function of time. (**a** SPRR1B, **b** KRT6B, **c** ICAM1, **d** COX7B)

Interestingly eight genes whose expression was significantly different according to the age or the interaction age/time were identified (Table 3), respectively, either because of different intensity of modulation as a function of the age of the donor (see SPPR1B in Fig. 3) or because of a different kinetics of modulation (see KRT6B in Fig. 3). They were mainly involved in keratinocyte differentiation.

**Table 3:**
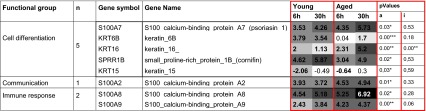
Eight genes that were significantly differentially modulated in young and aged skin

The mean fold change values at 6 and 30 h are reported at log 2 scale. Those with bold characters correspond to those that were considered as significantly modulated as defined in the methods. If there were >0.9 = up-regulation, or if <−0.6 = down regulation. A two-way Anova test was performed to compare aged and young groups. The significances of the age factor (a) and of the interaction of age on time (i) are reported

The shading darkens as the intensity of gene modulation increases

* Significant (Anova test, *p* value <0.05)

** Highly significant (Anova test, *p* value <0.01)

Furthermore, there were 186 genes which were modulated in the young population whereas they were not in the aged population, and inversely 198 genes were modulated in the aged population only. These two sets of genes should tell about the age-related specificities which are set up during epidermal repair. However, in order to eliminate the cases of genes considered as modulated in one group and not in the other one but whose fold change value in both populations is closed, we selected the core of these age specific modulated genes. We reduced the comparison to 6 and 30 h, because these two time points are those when most of the genes are modulated in the young and aged group, respectively (see Fig. 3). The mean distance *D* between two age groups was calculated for each gene as described in the methods. Genes with an inter group distance of at least *D* + 2sem were selected as those that showed the greatest difference between the age groups post TS. These genes were the most reliable and representative of the age-related specificities during epidermal repair. This second selection gave Table 4, composed of 23 genes that were specifically modulated in the young group and Table 5 composed of 40 genes that were specifically modulated in the aged group. Table 4 appeared to be enriched with genes involved in DNA, RNA management and protein processing while Table 5 appeared to be enriched with genes involved in cell communication, detoxification and oxidative stress management.

**Table 4:** 23 genes that were specifically modulated in young skin: The mean fold change values at 6 and 30 h are reported at log2 scale

Functional group	Total	Gene symbol	Gene name	Young		Aged		*p* values	
				6 h	30 h	6 h	30 h	6 h	30 h
Adhesion	3	ADAM15	A disintegrin and metalloproteinase domain 15 (metargidin)	**0.97**	0.48	0.03	0.69	0.06*	0.25
		CDH1	Cadherin 1, type 1, E-cadherin (epithelial)	**1.52**	0.40	0.56	0.32	0.01**	0.93
		ITGAM	Integrin, alpha M (complement component 3 receptor 3 subunit)	**1.74**	0.43	0.39		0.22	0.86
Cell cycle–growth–proliferation	1	KHDRBS1	KH domain containing, RNA binding, signal transduction associated 1	**1.56**	0.58	0.04	1.22	0.04**	0.4
Cellular matrix	1	TUBB4	Tubulin, beta 4	**1.94**	0.95	0.82	0.90	0.11	0.93
Communication	2	CAP1	CAP, adenylate cyclase-associated protein 1	**1.3**	0.31	0.18	0.7	0.02**	0.33
		IFNAR2	Interferon (alpha, beta and omega) receptor 2	**1.43**	0.47	0.53	−0.1	0.39	0.06*
DNA–RNA–transcription–translation	6	XRCC1	X-ray repair complementing defective repair in Chinese hamster cells 1	**1.25**	0.44	0.41		0.15	1
		H2AFB2	H2A histone family, member B2	**1.27**	0.81	0.14		0.05**	0.07*
		RNPS1	RNA binding protein S1, serine-rich domain	**1.43**	0.58	0.08	0.35	0.03**	0.76
		SAP18	Sin3A-associated protein, 18 kDa	**1.66**	0.95	0.79	0.79	0.02**	0.73
		EIF1	Eukaryotic translation initiation factor 1	**1.24**	0.45	−0.34	0.44	0.02**	1
		ARL4D	ADP-ribosylation factor-like 4D	**1.96**	0.24	0.29	0.1	0.06*	0.762
Immune response	3	IL3RA	Interleukin 3 receptor, alpha (low affinity)	**1.53**	0.61	0.67	−0.0	0.247	0.06*
		PTGES	Prostaglandin E synthase	**1.55**	0.46	0.57	0.86	0.03**	0.09
		ICAM1	Intercellular adhesion molecule 1	**1.46**	0.48	0.54	−0.2	0.22	0.03**
Transport	3	GLG1	Golgi apparatus protein 1	**1.04**	0.37	−0.29	0.15	0.03**	0.61
		GOLGA4	Golgi autoantigen, golgin subfamily a, 4	**1.18**	0.29	0.36	1.03	0.11	0.69
		SLC31A1	Solute carrier family 31 (copper transporters), member 1	**1.54**	0.32	0.4	−0.1	0.04**	0.11
Diverse (extracellular matrix)	1	MMP7	Matrix metalloproteinase 7	**0.93**	0.96	−0.03		0.07*	0.33
Diverse (lipid metabolism)	1	PLCL1	Phospholipase C-like 1	**1.58**	0.49	0.77		0.33	0.86
Diverse (melanin metabolism)	1	SILV	Silver homolog (mouse)	**1.41**	0.25	−0.03	0.58	0.06*	1
Diverse (nucleus envelope)	1	LMNA	lamin A/C	**1.77**	**1.06**	0.88	0.71	0.24	0.17

Those with bold characters correspond to those that were considered as significantly modulated as defined in the methods. If there were >0.9 = up-regulation, or if <−0.6 = down regulation. The young and aged data were compared at 6 and 30 h by mean of a Mann–Whitney test: * *p* value <0.1, ** *p* value <0.05

**Table 5:** 40 genes that were specifically modulated in aged skin based on D + 2sem: the mean fold change values at 6 and 30 h are reported at log2 scale

Functional group	Total	Gene symbol	Gene name	Young		Aged		*p* value	
				6 h	30 h	6 h	30 h	6 h	30 h
Apoptosis	1	DAPK3	Death-associated protein kinase 3	0.17	0.26	**−1.17**	−0.29	0.03**	0.55
Cell cycle, growth, proliferation	2	CDC37	CDC37 (cell division cycle 37, *S. cerevisiae*, homolog)	1.02	0.51	0.74	**1.56**	0.55	0.11
		CDK2AP1	Cyclin-dependent kinase 2-associated protein 1	−0.07	0.00	0.51	**1.57**	0.53	0.08*
Cell differentiation	1	MDK	Midkine (neurite growth-promoting factor 2)	0.68	0.50	0.53	**1.36**	0.66	0.01**
Cellular matrix	3	ARPC4	Actin-related protein 2/3 complex, subunit 4 (20 kDa)		0.11	0.84	**1.21**	0.286	0.04**
		NID1	Nidogen_(entactin)	−0.07	0.03	−0.06	**1.35**	0.429	0.01**
		GSN	Gelsolin_(amyloidosis,_Finnish_type)	1.35	0.04	−0.39	**−1.11**	0.01**	0.01**
Communication	9	ITPKC	Inositol 1,4,5-trisphosphate 3-kinase C	0.89	0.43	0.47	**1.33**	0.25	0.01**
		MAP3K5	Mitogen-activated protein kinase kinase 5 kinase 5^1^activated_protein_kinase_kinase_kinase_5	−0.18	−0.16	0.15	**1.01**	0.931	0.03**
		PIK3CD	Phosphoinositide-3kinase,_catalytic,_delta_polypeptide	−0.01	−0.04	0.15	**1.12**	0.43	0.03**
		PRKCG	Protein kinase C, gamma	0.05	−0.09	−0.02	**0.85**	0.25	0.02**
		FGA	fibrinogen, A alpha polypeptide	0.88	0.30	0.75	**1.70**	0.79	0.01**
		TLE4	Transducin-like enhancer of split 4, homolog of Drosophila E(sp1)	0.99	0.28	0.72	**1.47**	1	0.00**
		TSPAN3	Tetraspanin 3	0.86	0.70	0.46	**1.83**	0.11	0.11
		TRIO	Triple functional domain (PTPRF interacting)	1.49	0.42	0.79	**1.64**	0.33	0.11
		ANXA6	Annexin A6	1.02	0.37	0.59	**1.65**	0.43	0.18
Detoxification–oxidative stress	5	COX7B	Cytochrome *c* oxidase subunit VIIb	0.52	0.82	1.30	**1.96**	0.90	0.04**
		COX7C	Cytochrome *c* oxidase subunit VIIc	1.18	0.56	0.78	**1.46**	0.93	0.03**
		CYC1	Cytochrome *c*-1		0.49	0.63	**1.50**	1	0.04**
		UQCRQ	Ubiquinol-cytochrome *c* reductase, complex III subunit VII, 9.5 kDa	0.71	0.55	0.67	**1.69**	0.90	0.01**
		GPX3	Glutathione_peroxidase_3_(plasma)	−0.05	0.03	0.26	**1.09**	0.66	0.00**
DNA–RNA–transcription–translation	8	CBX3	Human heterochromatin protein_HP1Hs-gamma_mRNA	0.04	0.07	0.15	**1.55**	0.33	0.09*
		MCRS1	Microspherule protein 1	0.19	−0.03	0.46	**1.25**	0.54	0.03**
		POLR2G	Polymerase (RNA) II (DNA directed) polypeptide G	1.10	0.44	0.60	**1.52**	0.43	0.00**
		SF3B2	Splicing factor 3b, subunit 2, 145 kDa	1.23	0.56	0.78	**1.55**	0.55	0.02**
		SNRPA1	Small nuclear ribonucleoprotein polypeptide A′	0.70	0.23	0.83	**1.35**	1	0.04**
		SSBP1	Single-stranded DNA binding protein 1	1.16	0.43	**1.56**	**1.90**	0.25	0.02**
		SUMO2	small Ubiquitin-like Modifier type 2	0.01	−0.12	0.46	**1.51**	1	0.00**
		ESRRA	Estrogen-related_receptor_alpha	0.18	−0.05	0.12	**1.39**	0.66	0.08*
Energy	1	BPGM	2,3-Bisphosphoglycerate_mutase	0.17	−0.14	−0.04	**1.24**	0.43	0.05**
Immune response	2	TCRB	T-cell_receptor,_beta_cluster	0.02	0.05	0.65	**1.53**	0.33	0.17
		CLEC2B	C-type lectin domain family 2, member B	0.98	0.29	0.59	**1.39**	0.54	0.18
Lipids (barrier function)	1	GBA	Glucosidase,_beta;_acid	0.20	−0.14	0.26	**1.11**	0.79	0.01**
Proteolysis	2	PSMA5	Proteasome (prosome, macropain) subunit, alpha type, 5	1.16	0.62	**1.26**	**1.90**	1	0.05**
		PSME2	Proteasome (prosome, macropain) activator subunit 2 (PA28 beta)	0.81	0.70	0.87	**1.57**	0.90	0.11
Transport	1	ATP1A1	ATPase, Na +/K + transporting, alpha 1 polypeptide	0.65	0.61	0.81	**1.79**	0.63	0.02**
Diverse (oxygen transport)	1	NGB	Neuroglobin	0.94	0.44	0.28	**1.67**	0.13	0.04**
Diverse (steroid metabolism)	1	HSD17B10	Hydroxysteroid (17-beta) dehydrogenase 10	0.69	0.58	0.53	**1.53**	0.56	0.00**
Diverse (sugar binding)	1	LGALS2	Lectin,_galactoside-binding,_soluble,_2_(galectin_2)	0.14	−0.07	0.22	**1.34**	0.54	0.01**
Diverse (vit K metabolism)	1	PRRG2	Proline-rich_Gla_(G-carboxglutamic_acid)_polypeptide_2	−0.14	−0.08	0.03	**1.11**	1	0.01**

Those with bold characters correspond to those that were considered as significantly modulated as defined in the “Methods”. If there were >0.9 = up-regulation, or if <−0.6 = down regulation. The young and aged data were compared at 6 and 30 h by mean of a Mann–Whitney test: * *p* value <0.1; ** *p* value <0.05

## Discussion

The skin, especially the SC, is the human body’s first line of defense against external aggressions. Understanding the mechanisms implemented by normal epidermis to maintain epidermal integrity is crucial and may help discover new treatments intended for improving epidermal repair and homeostasis.

Our study aimed at comparing the kinetics of barrier function recovery after SC removal in young and aged volunteers. The purpose was to identify relevant, reproducible and significant biomarkers reflecting a possible age-related imbalance of barrier function. Most of the clinical trials described to date are based on TEWL measurements to analyze epidermal barrier function or measure the return of the epidermis to a normal homeostatic state after aggression. However, TEWL is measured using an evaporimeter including both a humidity and a temperature detector, which are submitted to numerous variation factors such as room temperature and hygrometry, air turbulence and even the state of mind of the volunteers [1, 24].

In a previous study that aimed to determine the effect of various cosmetic treatments on barrier function we performed a transcriptomic analysis of epidermis in addition to TEWL measurements [13]. The use of microarrays revealed reproducible transcriptomic markers that were common to the various treatments as well as markers that were specifically representative of each of the treatments, while TEWL measurements could not reveal differences between the treatments. Moreover, while TEWL is an overall measurement of barrier function, transcriptomic analysis gives insights into the understanding of the various biological functions underlying epidermal recovery process.

More recently, we went further in the use of transcriptomic analysis to better understand epidermal recovery process. A study which extended from 2 to 72 h following SC removal allowed us to describe the various biological functions involved in barrier recovery in a chronological way and consequently to characterize the sequence of cellular events taking place at each step of epidermal repair in young epidermis. Some of these results were confirmed later at the proteomic level [3]. These results formed a basis to better address unbalance that could occur and lead to disturbances in epidermal repair and homeostasis.

With aging some discomfort is likely to be linked to altered epidermal barrier permeability and epidermal homeostasis. To better understand the causes of these age-related events, in the present study we compared epidermal recovery in young and aged skin. Both TEWL measurements and transcriptomic studies were carried out at 2, 6, 18, 30 and 72 h after TS.

Our results highlighted differences in the capacity of young and aged epidermis to repair following SC removal. First, the assessment of barrier function recovery via TEWL measurements in young and aged epidermis showed a delay in the aged group (Fig. 1). Indeed, although similar at 72 h, the results showed that the recovery in the young group appeared to be dramatically significant during the overall kinetics, while there were no significant evolution until 30 h in the aged group. While basal TEWL is slightly decreased in elderly people compared to young people [10], the delay in barrier recovery has already been observed both in humans and mice [5, 6, 10, 11]. Indeed, Ghadially et al., studied human barrier recovery after sequential tape strippings, in young and aged human epidermis in vivo. Although experimental study designs were different therefore making difficult the comparison of the results with ours, similar conclusions were drawn. Barrier recovery was significantly higher in young epidermis compared to aged epidermis especially at time points 24, 48 and 72 h. One hundred and forty-four hours were necessary in aged group to reach the same barrier recovery level than in young group. It has to be noted that, in mice, barrier recovery after tape stripping was also delayed in aged vs. young epidermis, with a similar pattern to human epidermis but over a shorter time period [5, 10, 11].

Such a result confirmed a slower epidermal turn over and a less effective repair process in aged skin. This assumption is reinforced by the analysis of modulated genes distribution as a function of time. The highest rate of modulated genes was clearly found at 6 h in young group, whereas it was delayed to 30 h in aged group (Fig. 2). Thus cellular activity linked to the repair process may be engaged earlier in young epidermis than in aged epidermis.

Secondly, we observed striking differences in modulated genes according to age. Whereas about 400 genes were modulated in each age group only 184 were common to both groups and 198 and 186 genes were modulated only in the aged or in young group, respectively. Nevertheless, the overall biological functions supported by these genes were similar. For example, about 70 genes involved in DNA and RNA processing, synthesis and repair were modulated in each group (Table 2). However, in the young group most of them were modulated at 6 h, whereas in the aged group they were mostly modulated at 30 h. In fact, the molecular functions that were previously identified as early modulated after tape stripping in young epidermis [23] are mostly delayed in aged epidermis. It is mostly the case for those functions related to cell adhesion, oxidative stress management and cellular matrix constitution, processes that mediate intra- or inter-cell signaling, cell growth and proliferation and DNA or RNA processing, synthesis and repair. This was less evident for the functions related to cell differentiation which appear to be functions coming into play at later stage.

Lastly, despite the fact that the overall biological functions involved in barrier recovery in young and aged epidermis are ultimately similar, the expression profiles of three sets of genes clearly showed age-related differences in the fine tuning of epidermal response. A first set of eight genes was commonly modulated in both young and aged epidermis (Table 3), a second set of 23 genes was specific to young epidermis and a third set of 40 genes was specific to aged epidermis (Tables 4, 5).

The compilation of these results strongly suggests that one of the reasons for the delayed barrier recovery process in aged skin is the overall delay in gene response. Indeed, this includes a delayed induction of genes that are responsible for cell signaling, DNA transcription, RNA translation, but also genes that are important for barrier function recovery such as those involved in epidermal differentiation process.

As an interesting example, we noticed a significant delay in the induction of KRT6B gene in aged skin. Keratins constitute the intracellular intermediate filaments network of keratinocytes, and have an important structural function [9, 14, 19]. Keratinocytes express different types of keratin in specific conditions related either to the cellular stage of differentiation or to environmental challenges [8]. KRT6B is known to be strongly induced in keratinocytes after hyperproliferative stimuli such as wound healing, psoriasis, and other inflammatory disorders [15, 16, 25]. In 2003, Wong and Coulombe [25] proposed a model in which the KRT5/KRT14 pair of keratins that are expressed by keratinocytes in basal layers would provide keratinocytes a certain plasticity to facilitate cell migration and proliferation, whereas the KRT1/KRT10 pair of keratins should provide suprabasal keratinocytes a stronger mechanical resilience [19]. According to this model, the induction of the KRT6/KRT16 pair of keratins after injury would permit keratinocytes to satisfy both these conflicting needs, i.e., having sufficient cell malleability for migration and proliferation and sufficient resilience to survive the wound environment. When applied to our study, this model suggests that the induction of KRT6B and KRT16 would reflect a transient change in keratinocyte cytoskeleton in order to adapt to both the hyperproliferative stimulus triggered by TS and the need for sufficiently resilient cells to replace removed cells in suprabasal layers. The reported age-related delay in the induction of KRT6B might then, cause a delayed capacity of aged keratinocyte to adapt to injuries.

Another example is the significant difference in the induction of SPPR1B gene (cornifin) with an impressive up-regulation in young skin while the phenomenon is of less amplitude in aged skin with a delayed pic of induction (6 h in young skin versus 30 h in aged skin). In young skin, we had previously highlighted the surprising early and high activation of several genes involved in cornification such as cornifin, involucrin and small proline-rich protein 2C, whereas others like envoplakin or filaggrin were repressed [23]. We had hypothesized that the expression of some cornified envelope proteins may contribute to provide the epidermis with an emergency scaffold for barrier function recovery, facilitating the structural organization of the already available extracellular lipid matrix [4, 23]. The lower induction of cornifin in aged epidermis may reflect its poorer capacity to recover its barrier function.

In addition, other genes belonging to the epidermal differentiation complex (EDC), located on chromosome 1q21 were also differentially modulated such as S100A2, S100A7, S100A8 and S100A9 and to a lesser extent S100A10.

Some differences in the biological functions of modulated genes also allow understanding the molecular and cellular consequences of aging on epidermal repair. As an example, we observed in aged group an over-representation of genes involved in mitochondrial electron transport machinery (COX7B, COX7C, CYC1). This suggests that skin repair in aged skin requires more energy, which correlates with the high number of genes involved in the energy function that were modulated in aged skin (Table 2).

Altogether, the present study allowed us to identify specific transcriptomic signatures of epidermal repair in young and aged skin. These results provide a new way to describe epidermal repair and homeostasis. Moreover, transcriptomic analysis over time appeared to be more sensitive and informative than TEWL measurements to compare both young and aged epidermal repair. Indeed, significant differences were observed at 6 h in gene expression between young and aged skin, whereas at that time no differences were seen in barrier function recovery as assessed with TEWL. It would be interesting to complete these gene expression results at the protein level as already partially done [3]. However, this kind of clinical study requires a huge number of skin biopsies which may raise ethical concerns. Nevertheless, these findings should contribute to a finer efficacy assessment of a product or a process likely to improve epidermal renewal or homeostasis.
